# Regulating Monodispersity by Controlling Droplet Spacing

**DOI:** 10.1021/acs.langmuir.4c02058

**Published:** 2024-09-24

**Authors:** Dheeraj Sapkota, Laura L. A. Adams

**Affiliations:** Department of Physics and Astronomy, University of Minnesota - Duluth, Duluth, Minnesota 55812, United States

## Abstract

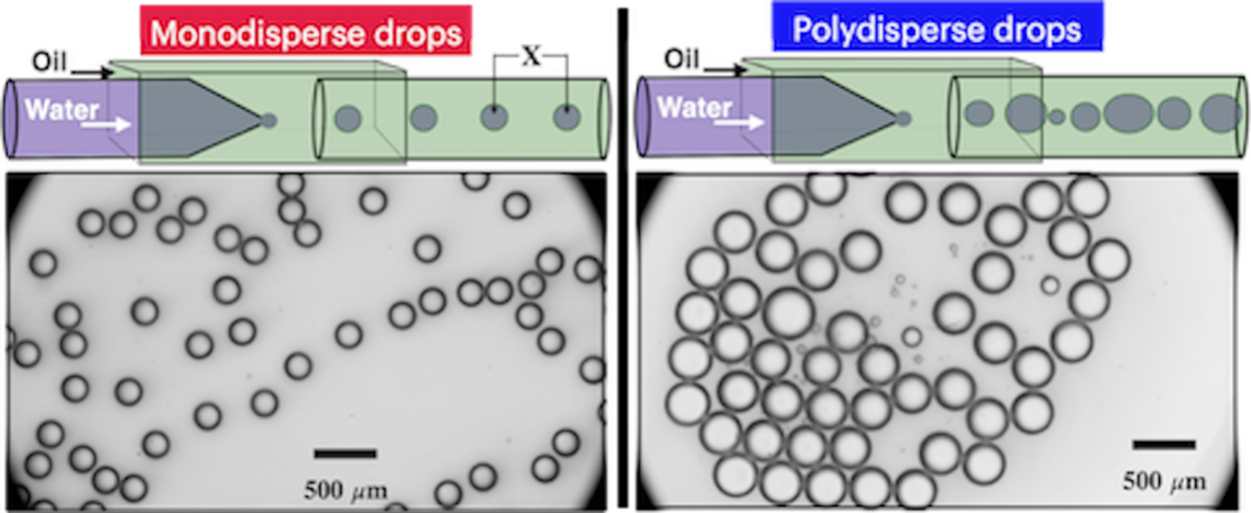

We report a new method for regulating monodispersity in the generation of single emulsions. The spacing between two consecutive emulsions during their generation is used to identify monodisperse and polydisperse regimes, with monodispersity having a size dispersion of <9% as an upper limit. A theoretical fit to our data is also presented. Moreover, a phase diagram of drop diameter as a function of inner and outer fluid flow rates indicates optimal flow parameters for the production of monodisperse drops. Our findings emphasize the robustness of using droplet spacing as a controlled parameter in regulating monodispersity, despite geometric differences in microfluidic devices.

## Introduction

Since its inception nearly 30 years ago,^1,2^ microfluidics has emerged as one of the main techniques for producing monodisperse single emulsions: drops of the same size.^3,4^ Single emulsions are drops of one fluid dispersed in another immiscible fluid, and not all single emulsions generated using microfluidics are created equal, that is, of equal size. As has been well documented, monodisperse emulsions can be generated with glass capillary devices when these devices are operating in the dripping regime.^5−7^ Polydisperse emulsions, on the other hand, are created when these devices are operating in the jetting regime. Typically, the distinction between these regimes is based on two dimensionless parameters: Weber number and capillary number.^8^ When both Weber and capillary numbers are relatively low and their sum is less than 1, the device is operating in the dripping regime, whereas jetting occurs when their sum is greater than 1.^9,10^

**Figure 1:**
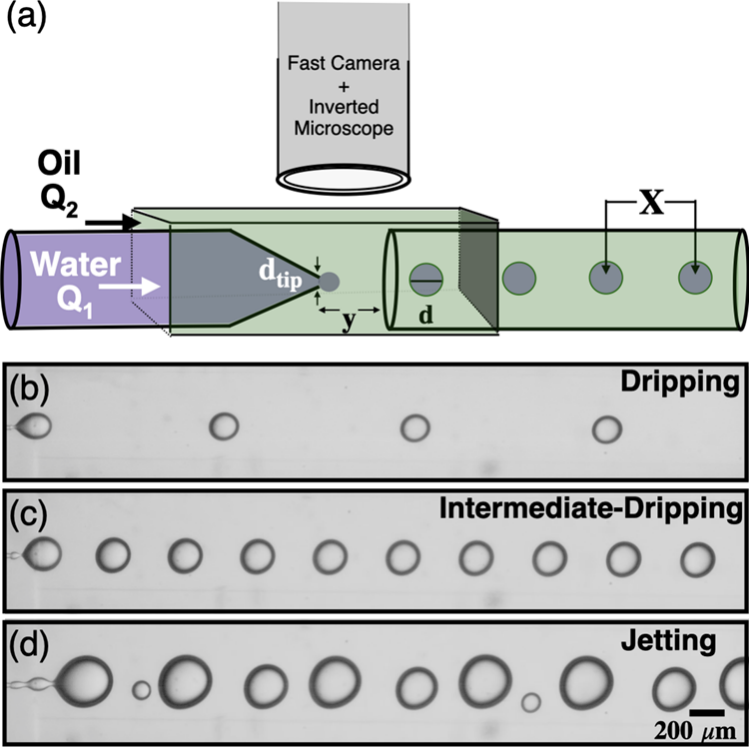
(a) Schematic of microfluidic device with a coflow geometry. (b–d) Fast camera images of single emulsions generated in the three regimes. The flow rates for the inner fluid *Q*_1_ are 100, 400, and 850 μL/h for (b), (c), and (d), respectively, with all regimes having the same outer fluid flow rate of *Q*_2_ = 4000 μL/h. These images were taken by using a frame rate of 3000 frames per second. The same scale bar applies to all fast camera images. Video of (b), (c), and (d) can be found on YouTube.^35^

The Weber number is the ratio between the inertial and surface tension forces, where the inertial force is quadratically proportional to the inner fluid’s velocity in a microfluidic device. The inner fluid is the fluid flowing through the inner tapered capillary as shown in Figure 1. The inertial force is relevant when the inner fluid velocity is high compared to the outer fluid velocity. The outer fluid is fluid flowing through the interstitial space between the tapered and square capillaries. Utada et al.^9^ define the Weber number as
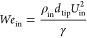
1where ρ_in_, *d*_tip_, γ, and *U*_in_ are the density of the inner fluid, the diameter of the tip orifice, the interfacial surface tension between the inner and outer fluids, and the velocity of the inner fluid, respectively.

In contrast to the Weber number, the capillary number relates the pull of the outer fluid, which is the drag or shear force, to the surface tension force, which seeks to keep the drop at the tip of the capillary. The capillary number is relevant when the device is operating in the dripping regime. Subsequently, these dimensionless numbers are defined in terms of flow velocities, interfacial tensions, relevant diameters, and viscosities.^9^

By measuring Weber and capillary numbers, we can theoretically predict the expected monodispersity-to-polydispersity transition. However, this requires instruments for determining fluid properties, e.g., viscosity and interfacial surface tension between two fluids. Without proper measuring tools, one must rely on textbook values for these fluid properties, which can vary depending on the manufacturer’s batch number or be altered with the addition of surfactants.^11^ Even with the correct viscosity and surface tension values, there is no universal agreement on which variables should be used when calculating the capillary and Weber numbers. For example, Utada et al.,^9^ Anna^12^ and Ren et al.^13^ define capillary number as
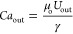
2where μ_o_, γ, and *U*_out_ are the viscosity of outer fluid, interfacial surface tension between inner and outer fluid, and the velocity of outer fluid, respectively. Erb et al.^14^ define capillary number as follows:
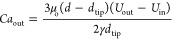
3where *d*, *d*_tip_, and *U*_in_ are the droplet diameter, the diameter of the tip orifice, and the velocity of the inner fluid, respectively. In Erb et al.’*s* definition, they use the modified version of Stokes formula for the shear force,^5^ where the term (*d* – *d*_tip_) reflects the nature of the coflow, where the cross-section of the inner capillary shields the drop from the continuous phase and the reduced relative velocity term (*U*_out_ – *U*_in_) accounts for the difference between the average speed of the continuous phase, and the average speed of the dispersed phase due to the forming droplet before pinchoff. There are also other definitions of capillary numbers for glass microfluidic devices that have been cited in the literature even though their essence remains unchanged.^15−17^ Furthermore, these variables in and of themselves can have large uncertainties depending on such things as the calibration of syringe pumps and tensiometers and the measurement of orifice and drop diameters.

Regardless of these challenges in measuring viscosity and interfacial surface tension and determining which version of Weber and capillary number to use, monodispersity and polydispersity are important properties in a wide range of applications. One application for monodisperse drops is in medicine, where monodisperse drops serve as drug carriers.^18−20^ Other current applications for monodisperse drops include micromotors,^21^ the fabrication of optical devices with liquid crystal drops,^22,23^ study of crystal growth^24,25^ and the use of monodisperse chemical reactants in cosmetic products.^26^ Polydisperse droplets, on the other hand, also have profound significance in research areas such as food science,^27−29^ foams,^30,31^ and combustion systems.^32^

In our work, we report an alternative and simpler method for determining the transition between monodispersity and polydispersity that does not rely on Weber and capillary numbers. By measuring an often overlooked parameter, droplet spacing, we can easily access all three hydrodynamic regimes, including the intermediate-dripping regime that lies between the dripping and the jetting regimes. We define droplet spacing as the spacing between two consecutive drops in the exit capillary, where the spacing between the last two consecutive drops is measured. This avoids the nonuniform fluid flow at the orifice.

## Experimental Section

### Materials

The inner fluid is filtered distilled water, and the outer fluid is pure vegetable oil with 10% (w/w) Abil EM 90 surfactant from Evonik Industries. Both the distilled water and vegetable oil are from Essential Everyday. The viscosity of oil–surfactant solution is measured with an Anton Paar rheometer and is found to be μ_o_ = 75 ± 3.5 mPa·s. The interfacial tension between water and oil is γ = 6.83 ± 1 mN/m as measured by the pendant drop method using an Ossila tensiometer.

### Microfluidics

To generate single emulsions, we use glass capillary devices in a coflow geometry^33,34^ where two immiscible fluids, oil and water, flow in the same direction as shown in Figure 1. All devices used here have the same configuration: two coaxially aligned cylindrical glass capillaries (World Precision Instruments, Inc., Sarasota, FL, 1B100-6) housed inside a square capillary (Atlantic International Technology, Inc., Rockaway, NJ, 810-9917). The cylindrical capillaries have an outer diameter of 1 mm, which almost matches the inner diameter (1.05 mm) of the square capillary. The tip of the inner capillary is tapered to a size of *d*_tip_ ≈ (22–32) μm using a micropipette puller (P-97, Sutter Instrument, Inc.), and the untapered exit capillary has an inner diameter *D*_c_ = 580 μm. The spacing between inner and exit capillaries, defined as capillary spacing, is not adjustable once the device is sealed with 5 min epoxy (Devcon, Danvers, MA). Each device has its own fixed capillary spacing, and for 9 devices, it ranges from 161 to 420 μm.

We recorded the generation of single emulsion drops within the microfluidic device using a 4× objective with a high speed camera (Phantom C321, Wayne, NJ). The inverted optical microscope we used is from Leica (DM IL LED Fluo). All of our experiments are performed at room temperature.

Fluids are introduced into microfluidic devices through PE/5 plastic tubing (Scientific Commodities, Inc., Lake Havasu City, AZ) connected to 20 mL luer-lock syringes (Millipore Sigma, catalog no. Z683620) with their flow rates controlled by Harvard PHD Ultra syringe pumps (Harvard Apparatus, Holliston, MA). In this work, the outer fluid flow rate, *Q*_2_, is set at a higher rate than the inner fluid flow rate, *Q*_1_.

## Results and Discussion

Like other research groups^9,13,36,37^ we observed three distinct regimes of drop formation: dripping, intermediate-dripping, and jetting, as shown in Figure 1b–d. Dripping always occurs at the lowest flow rate of inner fluid with a drop pinch-off exactly at the capillary’s orifice. Whereas in the intermediate-dripping regime, the drop pinches off from a narrow neck and not precisely at the orifice as shown in Figure 1c. The third regime, jetting, is defined as having the pinch-off from a widening and undulating neck that is longer than the previous regime. The jetting regime is distinct in that it also generates smaller satellite drops along with larger drops, as shown in Figure 1d.

**Figure 2:**
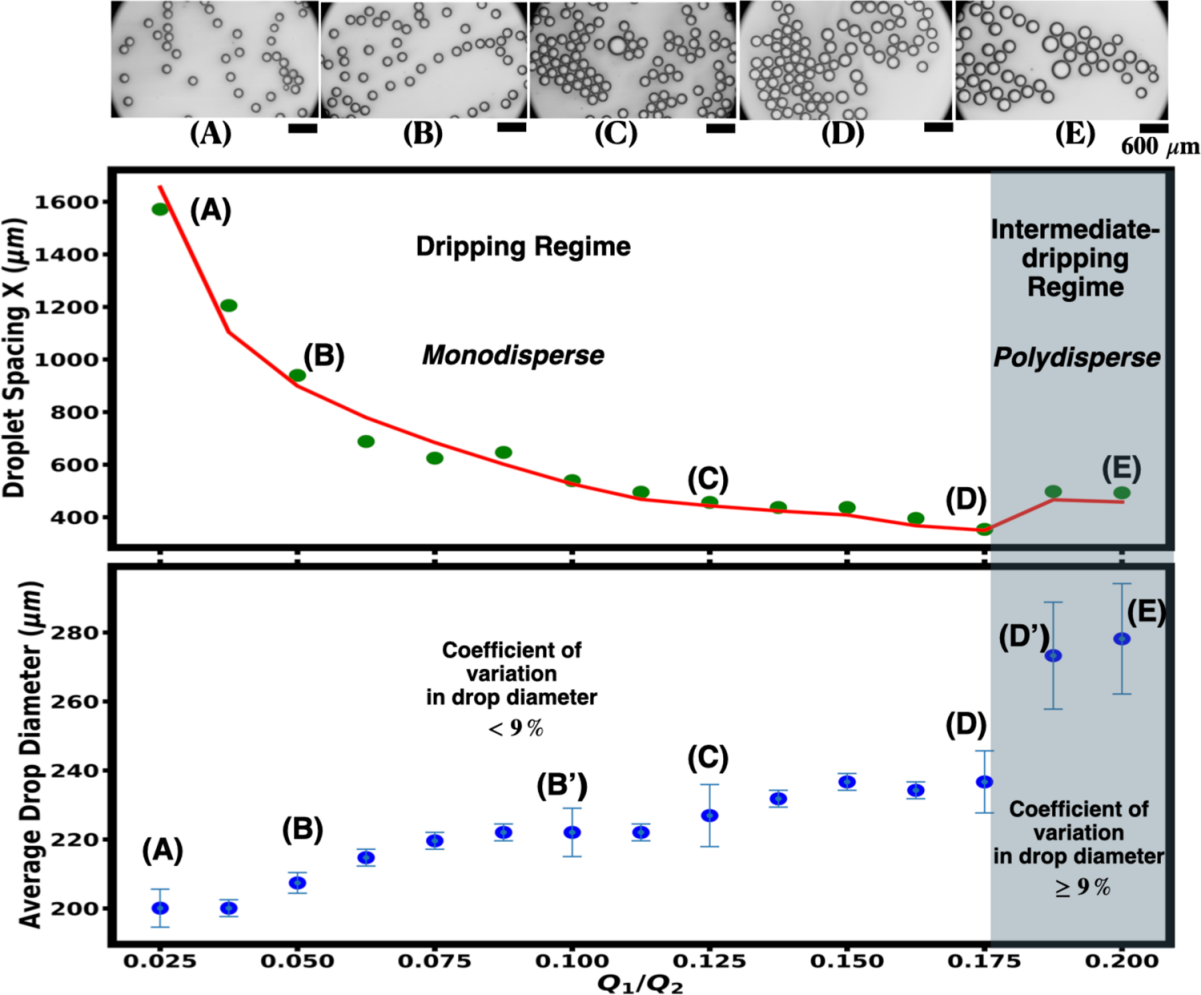
Top: optical microscope images with scale bars represent 600 μm. The large drop in image C is due to two drops coalescing and is responsible for a larger coefficient of variation than would be otherwise. Middle: Plot of droplet spacing vs *Q*_1_/*Q*_2_ with *Q*_2_ held constant at 4000 μL/h for device 1. Labels (A), (B), (C), (D), and (E) correspond to images above. The solid red line is a theoretical fit to the data as described in this paper. The error bars are ±4.88 μm and smaller than the symbol size. Bottom: Plot of the average drop diameter for the same data shown in the plot above it. The labeled data points have error bars from the standard deviation in measuring 80 droplets, while the unlabeled data points have error bars from systematic error ±4.88 μm.

Now we turn our attention to the focus of this paper: droplet spacing. By measuring the spacing between two consecutive drops as a function of flow rate ratios, *Q*_1_/*Q*_2_, we see a distinct jump between dripping and intermediate dripping regimes as shown in the top graph in Figure 2. As the inner fluid flow rate *Q*_1_ is slowly increased while maintaining a constant outer fluid flow rate *Q*_2_ of 4000 μL/h, the spacing between drops decreases until the device begins operating in the intermediate-dripping regime. Once in the intermediate-dripping regime, the drops’ spacing abruptly increases. This abrupt transition between dripping and intermediate dripping regimes is observed at a *Q*_1_/*Q*_2_ of 0.175 for this particular device (device 1). For device 1, the inner orifice size is 29 μm, and the spacing between the inner and outer capillaries for this device is 161 μm.

A jump in the droplet spacing has been observed for all nine glass capillary devices. This observation is irrespective of device geometric parameters, although where the jump occurs in terms of *Q*_1_/*Q*_2_ depends on device parameters as will be described below.

Moreover, by correlating droplet spacing with monodispersity, we observe that this jump is also a defining signature between monodisperse and polydisperse regimes. We collect at least 80 drops at five different *Q*_1_’s and with the same *Q*_2_. A representative sample of these 80 drops for five different *Q*_1_’s is shown in the top images of Figure 2. We measure their mean size, standard deviation, and coefficient of variation for each of the labeled points (A), (B), (C), (D), and (E) in Figure 2 and as tabulated in Table 1. Histograms of drop diameters for each labeled point (A), (B), (C), (D), and (E) are shown in Figures S1–S5. Furthermore, additional plots of droplet spacing versus *Q*_1_/*Q*_2_ along with histograms are shown in Figures S6–S14.

**Table 1:** Summary of Experimental Data is Given in Figure 2^a^

reference label	*Q*_1_/*Q*_2_	mean size *x̅* (μm)	standard deviation σ (μm)	coefficient of variation	regime
A	0.025	185 ± 5	11	5.95	dripping
B	0.050	195 ± 5	6	3.08	dripping
C	0.125	217 ± 5	18	8.29	dripping
D	0.175	224 ± 5	18	8.04	dripping
E	0.200	270 ± 5	32	11.85	interm. dripping

a The glass capillary device had a capillary spacing of 161 μm. The flow rate ratio, *Q*_1_/*Q*_2_, is the ratio of the inner and outer fluids, respectively. Diameters of more than 80 drops were measured from optical microscopy images using ImageJ software to arrive at the reported statistical values.

As expected, the average drop size increases with an increasing flow ratio *Q*_1_/*Q*_2_ as shown in the bottom graph of Figure 2. The coefficient of variation, calculated by taking the ratio of standard deviation to average drop size, fluctuates as the flow rate ratio increases. However, it never exceeds 9% before the jump, which occurs at (D) in the top graph of Figure 2. Furthermore, the coefficient of variation is less than 9% for drops in the dripping regime and greater than or equal to 9% for drops in the intermediate dripping regime, as listed in Table 1. Based on these observations, the jump in drop spacing, and the measurements of the coefficient of variation having a value less than 9%, we conclude that drop spacing determines the monodispersity and polydispersity regimes. The usual criterion for monodispersity is defined as having a coefficient of variation below 10%.^38−40^

To ascribe physics to our experimental findings, we developed a simple toy model to fit our data. If *t* is the droplet spacing time, that is, the time it takes for one drop to reach its neighboring drop, and *X* is the distance between centers of two consecutive drops, which is the droplet spacing, then the drop’s velocity is given by
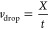
4The maximum frequency of drop formation just before jetting is,
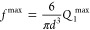
5where *d* is the drop diameter and *Q*_1_^max^ is the maximum inner-fluid flow rate before jetting.^15^ Rearranging terms and generalizing *Q*_1_ to represent all possible *Q*_1_ and not just the maximum *Q*_1_, the droplet pinch-off time *t*, which is the same as droplet spacing time, is
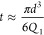
6 The droplet pinch-off time is the time it takes for the droplet to pinch-off from the orifice of the inner capillary after the previous droplet. Since drops move with an outer fluid, the drops’ velocity can also be defined as
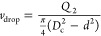
7where *D*_c_ is the inner diameter of the exit capillary.^14^ This drop velocity assumes that the flow is laminar and that the drop is moving at the centerline of the capillary.

From 4, 6, and 7, the droplet spacing is approximately:
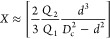
8and equal to
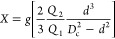
9where *g* is a dimensionless number containing two fitting parameters *y′* and *k* and is given by
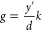
10These two fitting parameters *y′* and *k* are determined by a “trial and error” method that represents the best fit to the data. These values for nine devices are given in Table 2. Although the fitting parameter *k* has no known significance, the fitting parameter *y′* is comparable to the capillary spacing.

**Table 2:** Summary of Device Parameters and Fitting Parameters for Eq 10

devices	inner orifice size *d*_tip_ (μm)	capillary spacing *y* (μm)	fitting parameter *y′* (μm)	fitting parameter *k*	percent diff.
1	29 ± 2.5	161 ± 5	154	3.0	4.35
2	27 ± 2.5	381 ± 5	398	1.2	4.46
3	24 ± 2.5	224 ± 5	222	2.5	0.89
4	29 ± 2.5	207 ± 5	193	2.5	6.76
5	29 ± 2.5	171 ± 5	183	3.0	7.02
6	27 ± 2.5	420 ± 5	395	1.2	5.95
7	27 ± 2.5	161 ± 5	173	3.0	7.45
8	29 ± 2.5	264 ± 5	256	2.0	3.03
9	29 ± 2.5	281 ± 5	273	2.0	2.85

For all devices, the inner capillary orifices are all about 27 μm in diameter since these capillaries are tapered from the pipet puller with the same pulling parameters. However, if the inner capillary orifices are of a different size, we do not expect any major differences, other than the value at which the transition point occurs: this would either increase or decrease and thus alter the range in which one could get monodisperse droplets.

**Figure 3:**
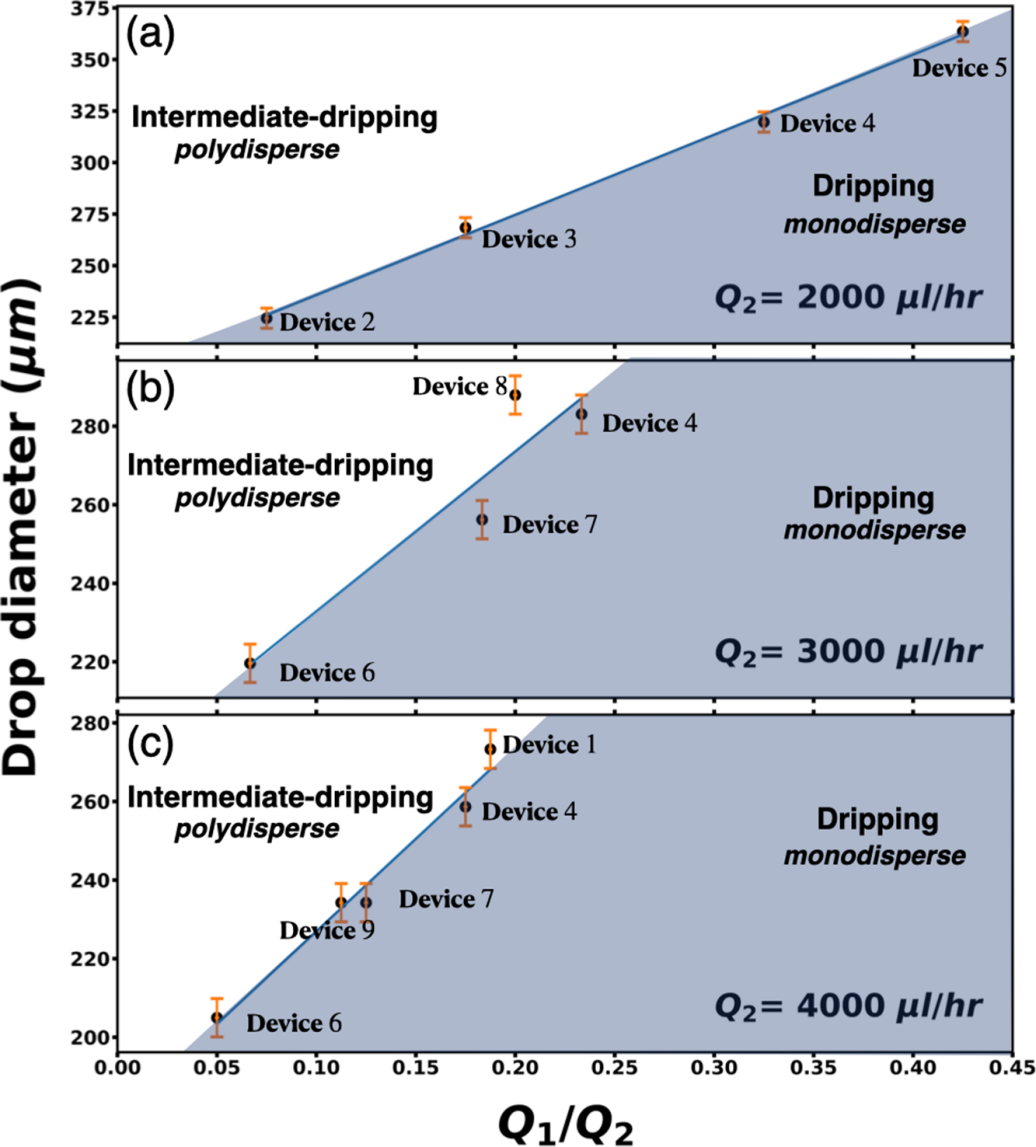
Phase diagram of average droplet diameter versus *Q*_1_/*Q*_2_ for three distinct outer flow rates (a) *Q*_2_ = 2000, (b) *Q*_2_ = 3000, and (c) *Q*_2_ = 4000 μL/h.

The experimentally measured capillary spacings *y* are in good agreement with best fit values *y′* as listed in Table 2. Furthermore, we observe that the higher the capillary spacing is, the lower the fitting parameter *k* is, and vice versa. The highest values of *y* are for devices 2 and 6, and the lowest values for *y* are for devices 1, 5, and 7. These *y* values play an important role in determining the best operational flow rates for monodisperse drops as the phase diagram demonstrates in Figure 3. A more detailed phase diagram is shown in Figure S15.

To correlate device parameters and flow rate ratios with the monodisperse drop regime, three-phase diagrams of average drop diameter versus flow rate ratio are plotted, with each diagram having a different *Q*_2_, as shown in Figure 3. Each point plotted in the phase diagram is the transition point determined by the jump in the spacing versus flow rate ratios as shown in the representative plot in the first graph in Figure 2. The capillary spacing *y* is the only device parameter varied. All other device parameters remain unchanged. For high *y* values, the system favors polydisperse drops even at low *Q*_1_ values. Thus, for optimal device operation for achieving monodisperse drops, *y* values should be small. This allows for a wider range of monodisperse drops.

Droplet spacing is also inversely proportional to the drop diameter: the smaller the spacing, the larger the drops. We determine the average drop diameter at each transition point, and in the phase diagram, we are plotting the droplet diameter instead of drop spacing. The phase diagram indicates that it is easier to generate smaller monodisperse drops than larger ones since the device operates in the dripping regime for a larger range of (*Q*_1_/*Q*_2_) values.

The transition between dripping and intermediate-dripping regimes for 9 devices, abet with three different outer-fluid flow rates *Q*_2_ = (2000, 3000, 4000) μL/h, showcases the breadth of the monodispersity regime. As *Q*_2_ increases, the regime of monodispersity increases. For example, our largest *Q*_2_, *Q*_2_ = 4000 μL/h, has the widest monodisperse regime, as indicated by the area of the gray region in the phase diagram. However, there is a limit to how high the outer flow rate can be and still be in the dripping regime: it should not be so high that the jetting regime is immediately reached.^9^

The slope and *y*-intercept values for each phase diagram are shown in Table 3. These values can be used to predict the average drop diameter (*d*) for a given flow rate ratio. This straight line equation in Table 3 also indicates that higher inner fluid flow rates *Q*_1_ result in larger diameter drops in the monodisperse regime. Importantly, steep slopes correlate to a wider dripping regime, which yields a larger range of monodisperse drops and is ultimately controlled by *Q*_2_ and capillary spacing.

**Table 3:** Straight Line Parameters: Slopes (*m*) and *y*-Intercepts (*c*) Corresponding to Figure 3^a^

the *d* in the equation represents the average drop diameter	
	*d* = *m*(*Q*_1_/*Q*_2_) + *c*
(a) at *Q*_2_ = 2000 μL/h	*m* = 389, *c* = 197 μm
(b) at *Q*_2_ = 3000 μL/h	*m* = 407, *c* = 192 μm
(c) at *Q*_2_ = 4000 μL/h	*m* = 471, *c* = 180 μm

a The *y*-intercept (*c*) correlates to the diameter of the drop at the minimum *Q*_1_ in the dripping regime. The slope (*m*) correlates with the range of the dripping regime.

## Conclusions

In conclusion, using glass capillary microfluidic devices for generating single emulsions, we found that droplet spacing is sufficient for determining monodispersity: the larger the spacing between drops, the more monodisperse the drops are. Our data-driven model with two fitting parameters accurately describes the response of droplet spacing with an increasing flow rate ratio of *Q*_1_/*Q*_2_. The phase diagram indicates the distinction between monodisperse and polydisperse drops. To get smaller monodisperse drops, the outer-fluid flow rate should be high, but not so high that the system starts jetting.^41^ Moreover, the system for generating monodisperse drops favors a small capillary spacing. A future endeavor would be to see if the droplet spacing and monodispersity correlation hold for generating single emulsions with different fluid properties and for double,^6^ multicomponent double,^42,43^ and higher-order emulsions.^44^

## Supporting Information Available

Additional experimental data including histograms for the coefficient of variation (COV) in Figure 2, plots of droplet spacing vs *Q*_1_/*Q*_2_ for Device 4 for three different *Q*_2_’s; plot of the droplet spacing versus *Q*_1_/*Q*_2_ for Device 3 showing the transition between dripping and intermediate dripping, the COV histograms for labeled data points in Device 3, and a detailed phase diagram (PDF)
