# Supplementary material for: Regulating Monodispersity by Controlling Droplet Spacing
Source: Langmuir. 2024 Sep 24;40(40):20938–44. doi: 10.1021/acs.langmuir.4c02058 (PMC11465740; doi:10.1021/acs.langmuir.4c02058)
Supplement: Supplementary file 1 — la4c02058_si_001.pdf [file la4c02058_si_001.pdf]

Supporting Information

Regulating Monodispersity by Controlling Droplet Spacing

Dheeraj Sapkota and Laura L. A. Adams*

Department of Physics and Astronomy, University of Minnesota - Duluth, Duluth, Minnesota 55812

*To whom correspondence should be addressed: Laura L. A. Adams (lladams@d.umn.edu)

This PDF file includes: Figures S1-S15.

Contents

S1- S5: Histograms of droplet diameter for labeled data in Figure 2.

S6-S8: Plots of droplet spacing vs Q_1/Q_2 for Device 4 at $Q_2 = 2000\mu$l/hr, 3000 μl/hr, and 3000 μl/hr.

S9: Plot of droplet spacing vs Q_1/Q_2 for Device 3 at $Q_2 = 2000\mu$l/hr.

S10-S14: Histograms of droplet diameter for labeled data points in Figure S9.

S15: Detailed phase diagram for all data not included in Figure 3.

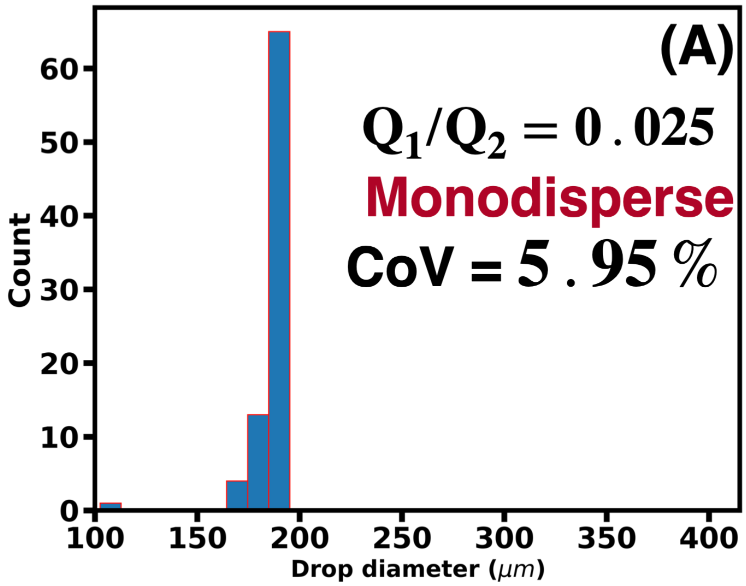

S 1: Histogram of droplet diameters for the data point A in Figure 2 and reference label A in Table 1. The coefficient of variation (COV) is 5.95%.

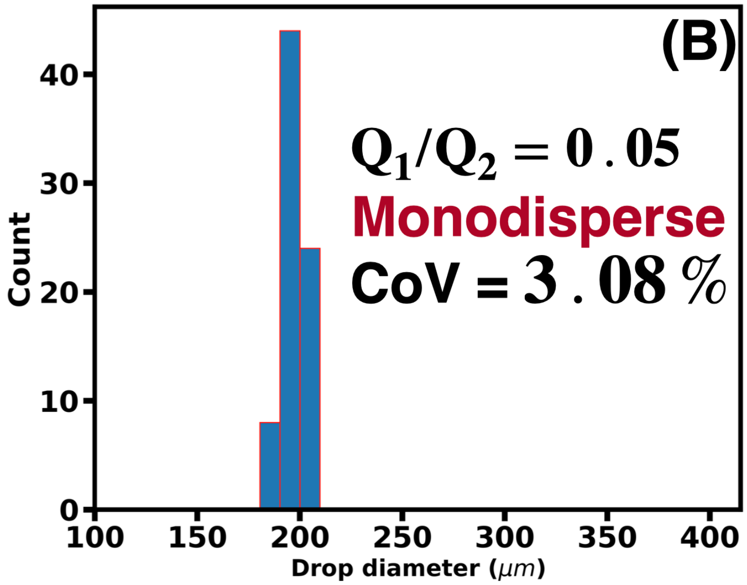

S 2: Histogram of droplet diameters for the data point B in Figure 2 and reference label B in Table 1. The coefficient of variation (COV) is 3.08%.

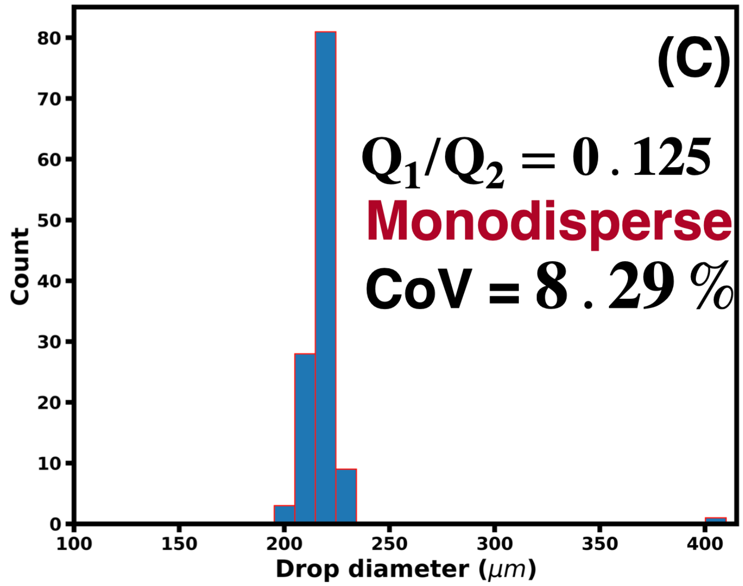

S 3: Histogram of droplet diameters for the data point C in Figure 2 and reference label C in Table 1. The coefficient of variation (COV) is 8.29%.

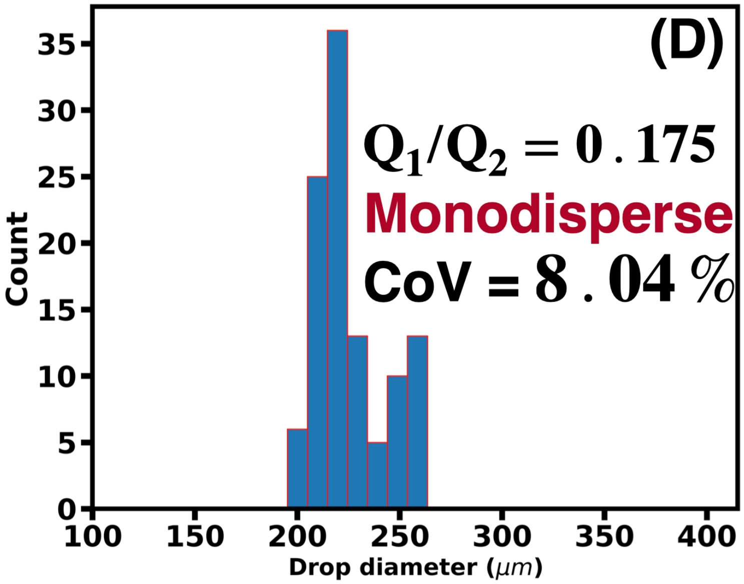

S 4: Histogram of droplet diameters for the data point D in Figure 2 and reference label D in Table 1. The coefficient of variation (COV) is 8.04%.

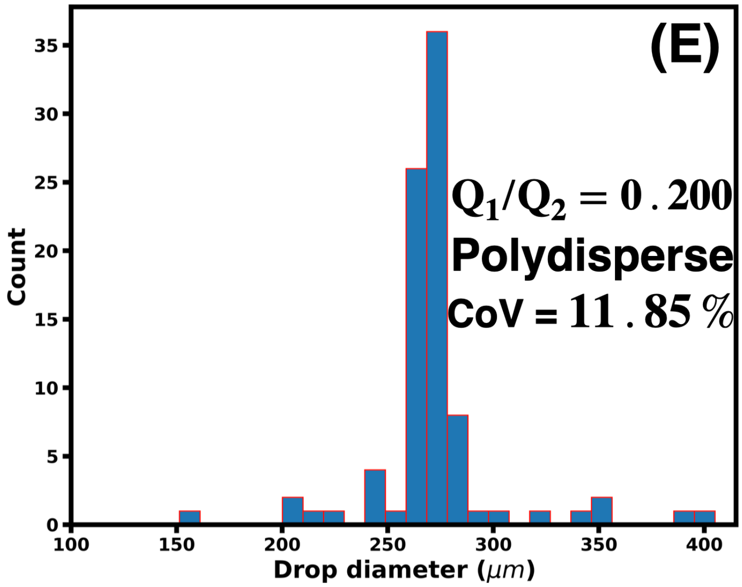

S 5: Histogram of droplet diameters for the data point E in Figure 2 and reference label E in Table 1. The coefficient of variation (COV) is 11.85%.

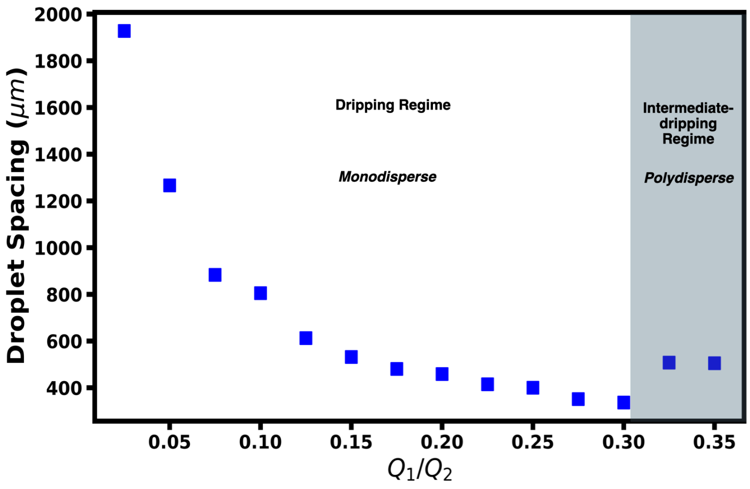

S 6: $Q_2 = 2000\ \mu l/hr$, Device 4, capillary spacing $y = 207\ \mu m$.

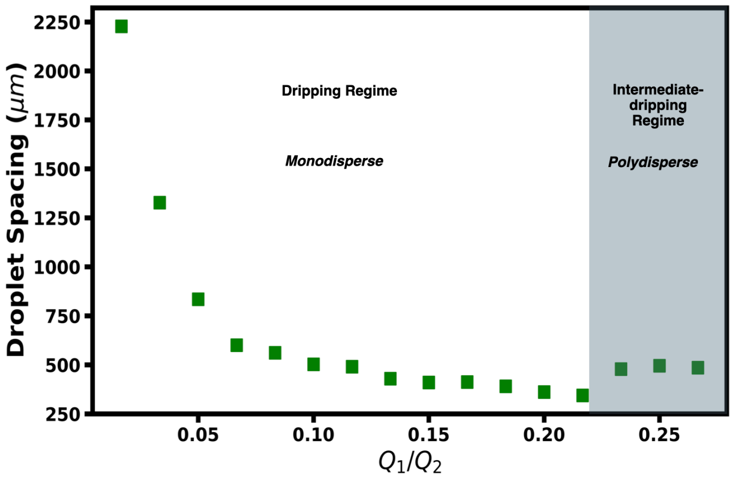

S 7: $Q_2 = 3000\ \mu l/hr$, Device 4, capillary spacing $y = 207\ \mu m$.

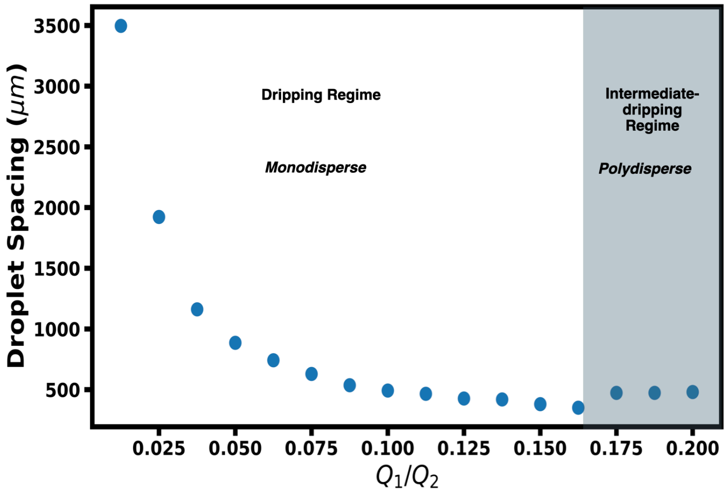

S 8: $Q_2 = 4000\ \mu l/hr$, Device 4, capillary spacing $y = 207\ \mu m$.

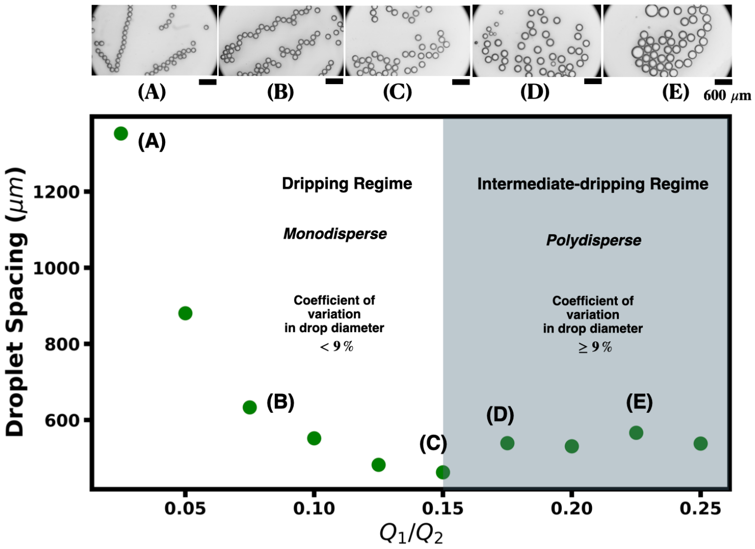

S 9: $Q_2 = 2000\ \mu l/hr$, Device 3, capillary spacing $y = 224\ \mu m$.

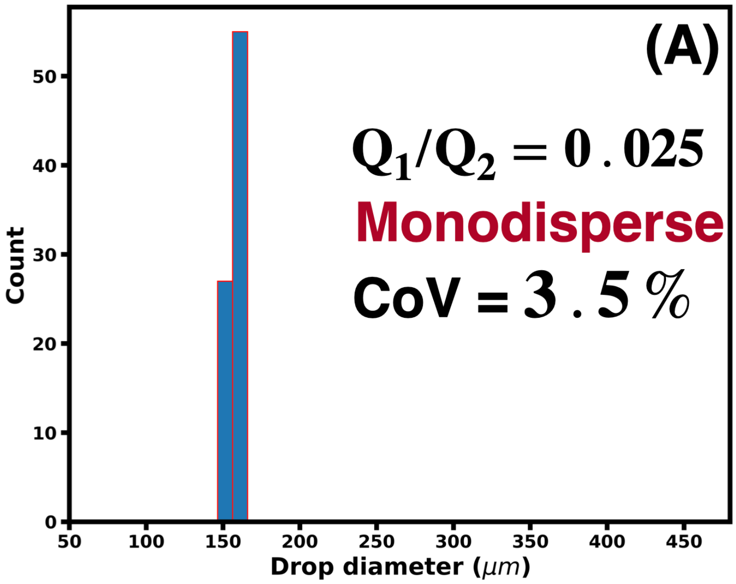

S 10: Histogram of droplet diameters for the data point A in Figure S9.

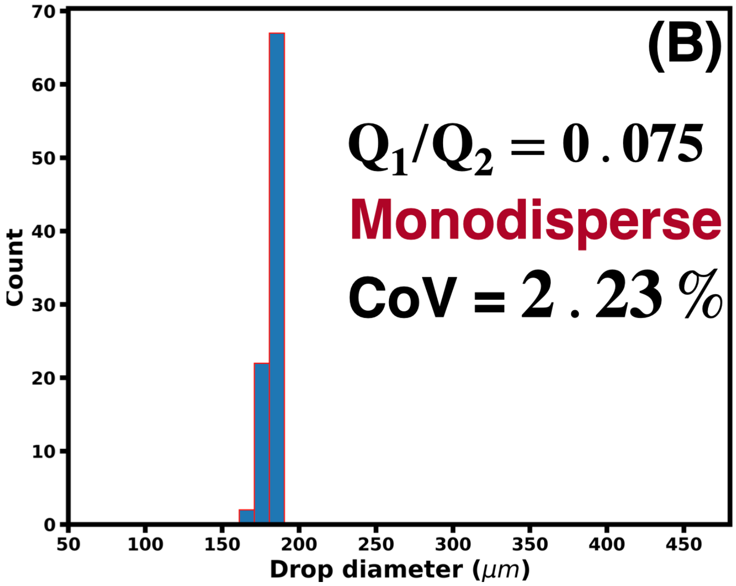

S 11: Histogram of droplet diameters for the data point B in Figure S9.

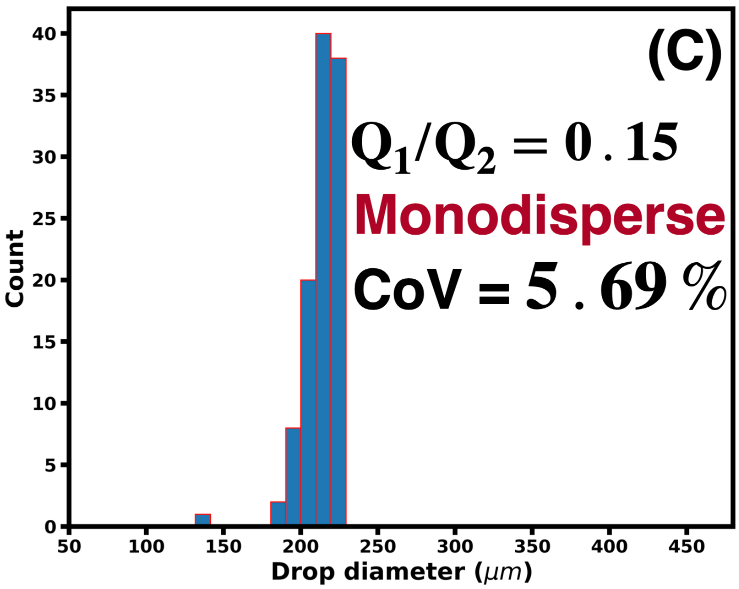

S 12: Histogram of droplet diameters for the data point C in Figure S9.

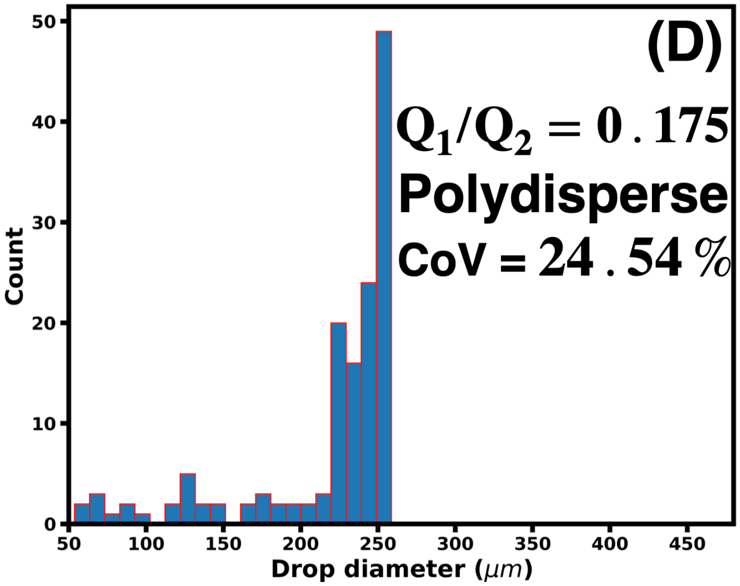

S 13: Histogram of droplet diameters for the data point D in Figure S9.

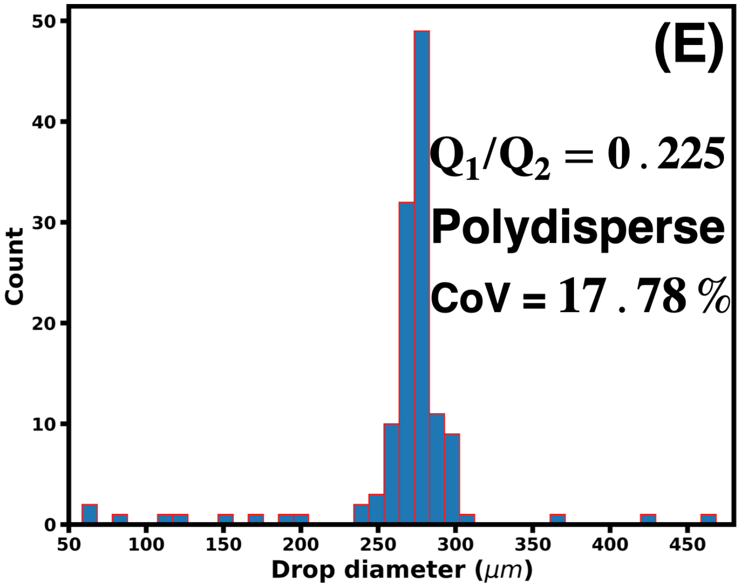

S 14: Histogram of droplet diameters for the data point E in Figure S9.

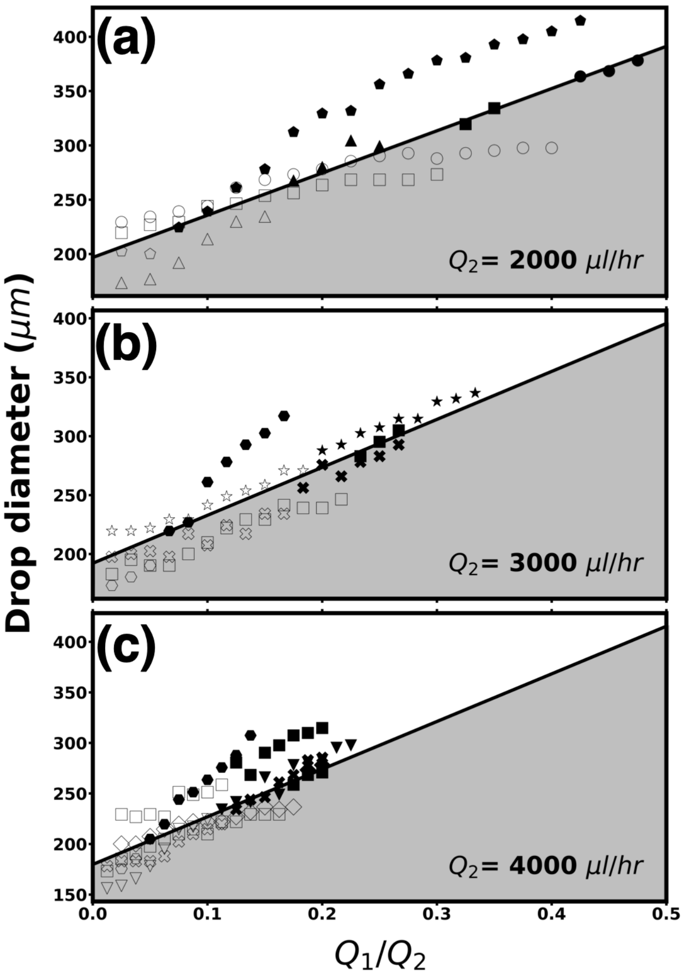

S 15: Details of the phase diagram. This figure includes all data that are not included in Figure 3. Unfilled symbols represent the dripping regime and the filled symbols represent the intermediate-dripping regime. Each symbol corresponds to different devices; Diamond: Device 1, Pentagon: Device 2, Triangle up: Device 3, Square: Device 4, Circle: Device 5, Hexagon: Device 6, X: Device 7, Star: Device 8, and Triangle down: Device 9. Linear lines are plotted to separate the dripping regime from the intermediate-dripping regime. Each line passes through the first point of the intermediate dripping regime. The slopes of these linear fitting lines are the same as that tabulated in Table 3. Devices are operating at (a) $Q_2 = 2000\mu l/hr$ (b) $Q_2 = 3000\mu l/hr$ (c) $Q_2 = 4000\mu l/hr$.
